# Surges in volcanic activity on the Moon about two billion years ago

**DOI:** 10.1038/s41467-023-39418-0

**Published:** 2023-06-22

**Authors:** Heng-Ci Tian, Chi Zhang, Wei Yang, Jun Du, Yi Chen, Zhiyong Xiao, Ross N. Mitchell, Hejiu Hui, Hitesh G. Changela, Tian-Xin Zhang, Xu Tang, Di Zhang, Yangting Lin, Xianhua Li, Fuyuan Wu

**Affiliations:** 1 Key Laboratory of Earth and Planetary Physics, Institute of Geology and Geophysics, Chinese Academy of Sciences, 100029 Beijing, China; 2 National Space Science Center, Chinese Academy of Sciences, 100190 Beijing, China; 3 State Key Laboratory of Lithospheric Evolution, Institute of Geology and Geophysics, Chinese Academy of Sciences, 100029 Beijing, China; 4 Planetary Environmental and Astrobiological Research Laboratory, School of Atmospheric Sciences, Sun Yat-sen University, 519080 Zhuhai, China; 5 State Key Laboratory of Mineral Deposits Research and Lunar and Planetary Science Institute, School of Earth Sciences and Engineering, Nanjing University, 210023 Nanjing, China; 6 School of Mining and Metallurgical Engineering, National Technical University of Athens, Athens, Greece; 7 Deep Space Exploration Laboratory, 100043 Beijing, China; 8 Lunar Exploration and Space Engineering Center, China National Space Administration, 100190 Beijing, China

**Keywords:** Geochemistry, Petrology

## Abstract

The history of mare volcanism critically informs the thermal evolution of the Moon. However, young volcanic eruptions are poorly constrained by remote observations and limited samples, hindering an understanding of mare eruption flux over time. The Chang’e-5 mission returned the youngest lunar basalts thus far, offering a window into the Moon’s late-stage evolution. Here, we investigate the mineralogy and geochemistry of 42 olivine and pyroxene crystals from the Chang’e-5 basalts. We find that almost all of them are normally zoned, suggesting limited magma recharge or shallow-level assimilation. Most olivine grains record a short timescale of cooling. Thermal modeling used to estimate the thickness and volume of the volcanism sampled by Chang’e-5 reveals enhanced magmatic flux ~2 billion years ago, suggesting that while overall lunar volcanic activity may decrease over time, episodic eruptions at the final stage could exhibit above average eruptive fluxes, thus revising models of lunar thermal evolution.

## Abstract (web summary)

This work estimates the eruption volume of the young Chang’e-5 lunar samples using diffusion chronology and thermodynamic simulations, and finds that there was an increase in volcanic eruption flux about 2.0 billion years ago.

## Introduction

There is agreement that volcanic activity on the Moon is temporally and spatially tied to the heat-producing elements^1^, unlike magmatic systems on Earth where more factors such as crustal recycling can trigger volcanic eruptions. Mare volcanism was most pronounced about 3.8–3.3 billion years ago (Ga) and then declined or disappeared by 2.9–2.8 Ga, as shown by the age distribution of the Apollo, Luna, and meteorite collections^2^. Such a duration of basaltic volcanism on the Moon is broadly consistent with models of thermal evolution^3,4^. However, recent studies of samples returned from the Chang’e-5 mission directly date lunar volcanism to 800–900 million years (Myr)^5,6^ later than previously measured in returned samples and meteorites^7,8^, where heat-producing elemental and water concentrations were lower than expected^6,9,10^, thus challenging the common view of lunar magmatism and thermal evolution of the Moon. Knowledge of the eruptive fluxes in terms of volume/mass could place constraints on this late-stage lunar volcanic activity. Although previous studies using remote sensing data investigated volcanic flux for young volcanic activity (<2.8 Ga; refs. ^11–13^), there are several complications, including: (i) the large uncertainties in crater counting chronology between 3 and 1 Ga (refs. ^14,15^); (ii) difficulties in the recognition of flow fronts caused by impact bombardment and other continuous erosional processes;^16^ and (iii) estimating the volume of a mare unit of a certain age, which is limited by insufficient knowledge of crater scaling laws and/or empirical equations of crater morphology^17^.

Here, we focus on using the chemical compositions and zoning patterns in olivine and clinopyroxene crystals from a large set of Chang’e-5 basalts with different textures to reconstruct their thermal history, estimate volcanic fluxes on the Moon, and discuss the implications this has for the most recent volcanism on the Moon. The 2-billion-year-old Chang’e-5 basalts from the northeastern Oceanus Procellarum terrane^5,6^ provide a unique opportunity for this. An effective method is to determine post-eruption lava flow cooling timescales using diffusion chronometry, which models diffusive relaxation of compositional boundaries within zoned minerals^18–21^. Extrapolated timescales can be used to estimate the thickness and volume of basaltic lava flows^22,23^. Applying these methods to the Chang’e-5 basalts and compiling previous results of eruptive flux throughout lunar history, we are able to identify that, despite its long-term secular cooling, the Moon was still capable of significant pulses of magmatism at about 2 Ga.

## Results

### Crystal classification and chemical zoning

Overall, the individual olivine and clinopyroxene grains in the Chang’e-5 basalts are relatively simple in high-resolution BSE images and do not exhibit any complex zonation (Supplementary Figs. 1–6; refs. ^5,6,9,10^). Four typical textures (porphyritic, subophitic, poikilitic, and equigranular) were identified in the basaltic rock clasts. Plagioclase and clinopyroxene are the predominant phases in the rock clasts with minor ilmenite and olivine grains^10^. In this work, we selected 21 olivine and 17 clinopyroxene crystals with sizes greater than 50 µm from 26 basalt clasts with different textures (sample no. CE5C0100YJFM00103, CE5C0400YJFM00406 and CE5Z0303YJ) and obtained 22 and 17 zoning profiles of major and minor elements for olivine and clinopyroxene, respectively (Supplementary Data 1 and 2). In addition, four clinopyroxene grains from ref. ^10^. were also added to the dataset.

**Fig. 1:**
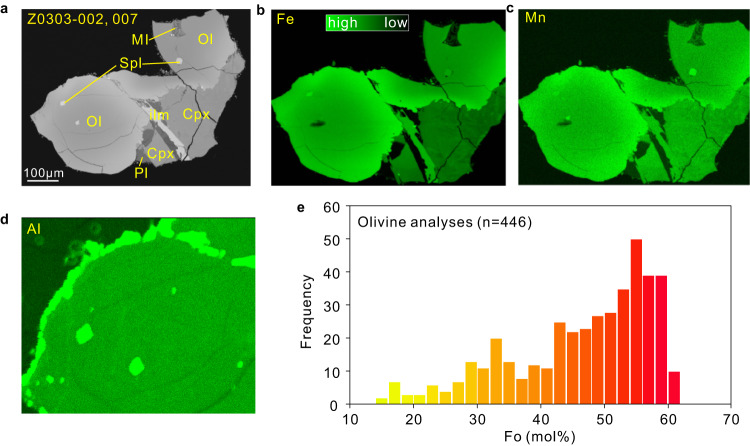
Representative olivine crystal and chemical composition in the Chang’e-5 basalt clasts. **a**–**d** High-resolution backscattered electron (BSE) image and Fe, Mn, and Al X-ray maps of representative olivine in the Chang’e-5 basalt clast (Z0303-002, 007). **d** is the enlarged image of the olivine crystal. The olivine grain shows nearly concentric compositional zoning with a core low Fe, low Mn, but Al is almost homogeneous from the core to the rim. Abbreviations: Ol olivine, Pl plagioclase, Cpx clinopyroxene, ilm ilmenite, Spl spinel, MI melt inclusion, Fe iron, Mn Manganese, Al Aluminum. **e** Frequency distribution of forsterite (abbreviated as Fo) composition [defined as 100 × Mg/(Mg + Fe), mole fraction] based on >400 analyses (this study and ref. ^10^).

**Fig. 2:**
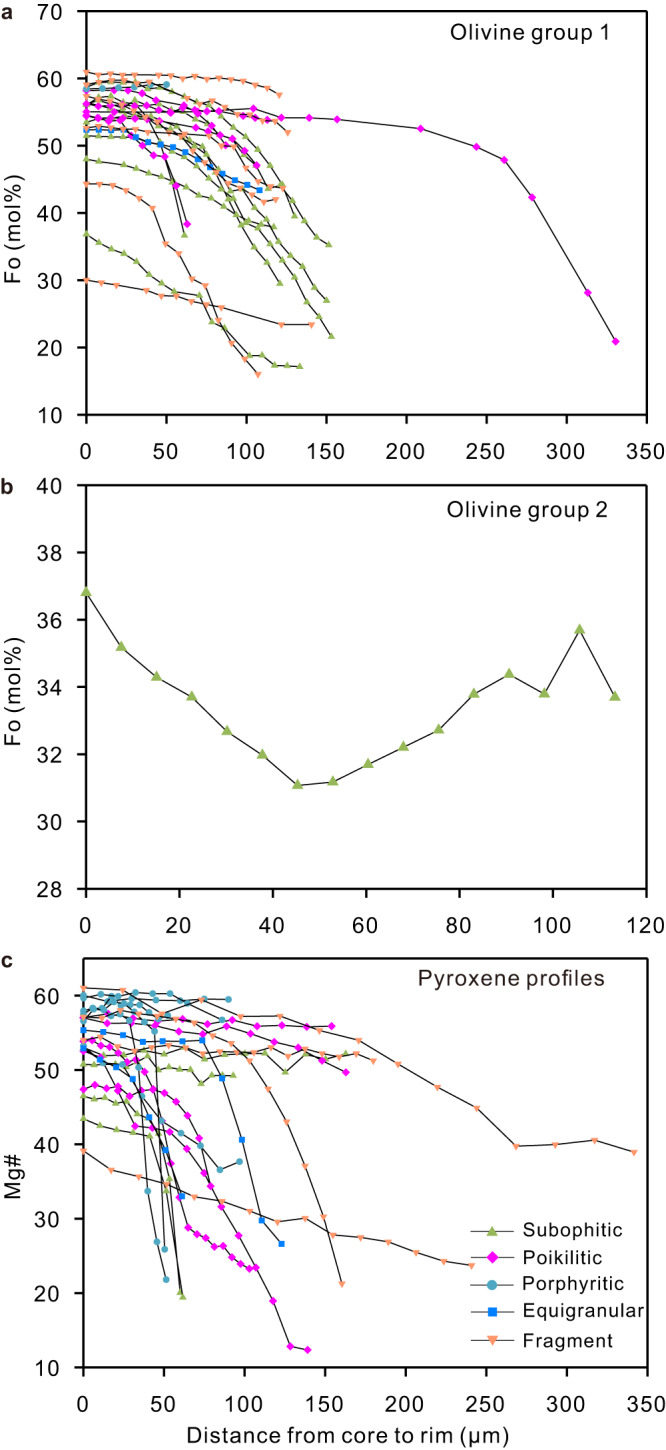
Chemical composition of olivine and clinopyroxene from Chang’e-5 basalt clasts. **a** Electron microprobe Fo (mol%) traverses for Group 1 olivine from core to rim. **b** A reverse profile for Group 2 olivine. **c** Electron microprobe Mg# [defined as 100 × Mg/(Mg + Fe), mole fraction] traverses for clinopyroxene from core to rim. EMPA traverses are marked in Supplementary Figs. 1–6, and data are provided in Supplementary Data 1–2. Abbreviations: Fo forsterite.

Qualitative X-ray maps of several olivine grains show nearly concentric zoning for major and minor elements (Fig. 1 and Supplementary Fig. 7), suggesting that the influence from subsolidus re-equilibrium is limited. The olivine crystals overall exhibit a wide range of forsterite (Fo), ranging from Fo_61.0_ down to Fo_10_ (Fig. 1e). According to the Fo variation from the core to the rims, olivine grains in the Chang’e-5 basalts are divided into two groups. Group 1 includes olivine with normal/no chemical zonation, while Group 2 refers to olivine with reverse chemical zonation. In combination with the Fo profiles published in ref. ^10^, we find that most of the profiles (21 out of 22) are normally zoned and the Fo variation for a single crystal from the core to the crystal rims is up to 35 (Fig. 2a). One olivine crystal (103-001, 007) is homogeneous in composition from core to rim (i.e., Fo_58.4–59.1_; Fig. 2a), but has a very thin (2–5 µm) Fe-rich rim (Supplementary Fig. 8). This may be growth-related and linked to the final rapid quenching of the magma^10^. In contrast, reverse zoning (Group 2) was observed only in one olivine crystal from sample 103-025, 009, where Fo values decrease from 36.8 at the rim to 31.1 in the core (Fig. 2b). This sample consists primarily of plagioclase and olivine, with minor amounts of clinopyroxene, ilmenite, and K-rich mesostasis (Supplementary Fig. 2).

All of the clinopyroxene crystals from the basalt clasts are normally zoned (Fig. 2c), with Mg# values [defined as 100 × Mg/(Mg + Fe), mole fraction] varying from 12.4 to 61.0 (Supplementary Data 2). The compositional range of clinopyroxene is nearly identical to that of forsterite. Clinopyroxene from porphyritic samples has very thin (<5 µm) Fe-rich rims (the relatively bright rims shown in Supplementary Fig. 8), also probably related to the late-stage rapid quenching of the magma. The olivine and clinopyroxene concentration profiles indicate a signature of highly evolved magma, as suggested by the overall low Fo values of the olivine crystals (Fig. 1e) and the low Mg# values of the clinopyroxene crystals (Fig. 2c). Note that some profiles (particularly in the subophitic grains) do not appear to have a concentration plateau in their cores, suggesting that they may have lost their core composition (Fig. 2).

### Cooling time of the lava flow retrieved from olivine crystals

**Fig. 3:**
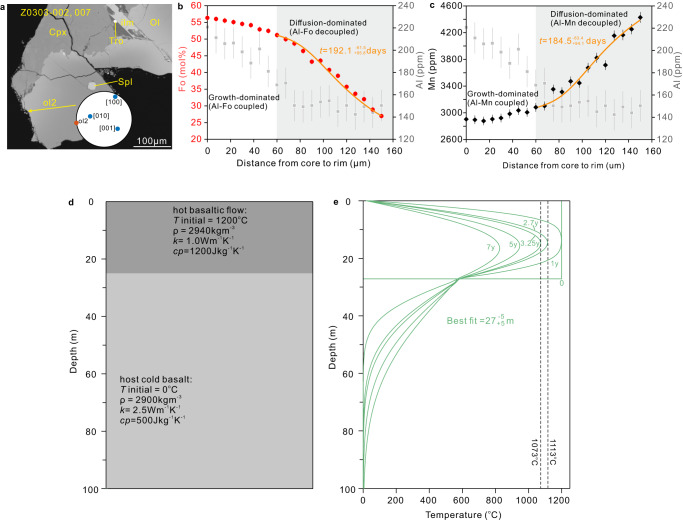
Representative zoning patterns in olivine, DIPRA results, and estimated thickness of the lava flow. **a** The BSE image of olivine. The arrow marks the positions of the electron microprobe line scan. Inset is an equal-area pole figure of the crystal showing the orientation of the main crystallographic axes (blue points) relative to the profile (shown as a red point). Abbreviations: Ol olivine, Cpx clinopyroxene, ilm ilmenite, Spl spinel, Tro troilite. **b**, **c** Fo, Al, and Mn contents and modeled profiles for sample Z0303-002, 007. The orange curves show the modeled fit for Fo and Mn calculated using the DIPRA software (see Methods for more details). Note that this profile contains a “coupled” and an “uncoupled” part, suggesting a two-stage crystallization history. We have only calculated the timescale of the “uncoupled” part here, as the growth time of the “coupled” part is generally negligible. The initial concentrations for the modeling are given in Supplementary Data 3. The error bar represents 1 standard deviation for each point, which are given in Supplementary Data 1. Abbreviations: Fo forsterite, Mn Manganese, Al Aluminum. **d** Simple one-dimensional modeling of conductive cooling of a basaltic flow. Depth and initial parameters for the underlying cold basalt and overlying high-temperature lava flow (*T* temperature, *ρ* density, *k* thermal conductivity, *cp* heat capacity) are shown. **e** Graph of temperature versus depth showing cooling curves at different depths after different times. The green curves are the modeling results for sample Z0303-002, 007. Assuming that the soil clasts are from the slowest cooling location, we obtain a minimum value for the thickness of the lava flow when the time period for the sample to cool from the olivine Fo core temperature to the rim temperature is equal to the timescale estimated for the same olivine grain.

Mineral zonation could be caused by a range of magmatic processes such as fractional crystallization, element diffusion after crystallization, magma mixing, and recharge events^24–26^. Aluminum is a slow diffusing element in olivine and has been shown to be immobile compared with Fe-Mg over the timescales of interest^27,28^. Thus, Al associated with Fe-Mg variations can distinguish between chemical zoning patterns formed either by crystal growth or by diffusion (following the approach outlined in refs. ^21,29,30^). Of the 21 olivine crystals from which Al content was measured, 6 crystals show an approximately linear correlation between Fo and Al concentrations, indicating growth-dominated chemical zonation. In the other 15 crystals, the Al content is almost unchanged within the uncertainty toward the rims, indicating diffusion-dominated zoning (Fig. 3a–c). This means that the whole olivine grains are classified into two types: diffusion-dominated or growth-dominated groups (Supplementary Table 1). The DIPRA software was originally developed for modeling the diffusion of multiple elements (e.g., Fe-Mg, Mn, Ni) in olivine and thus can be used to determine the timescale of diffusion (see Methods). Here, we used the model to calculate the times by fitting the measured Fe-Mg and Mn concentrations across the olivine, which most likely reflects the cooling times of the erupted magma. The diffusion model parameters and modeling results can be found in Supplementary Data 3. For clinopyroxene crystals, Mg and Fe content generally constrains magmatic evolution with timescales greater than 5 years (see Fig. 2 in ref. ^26^), and thus they are used here only to determine whether there is reverse or complex zonation.

**Fig. 4:**
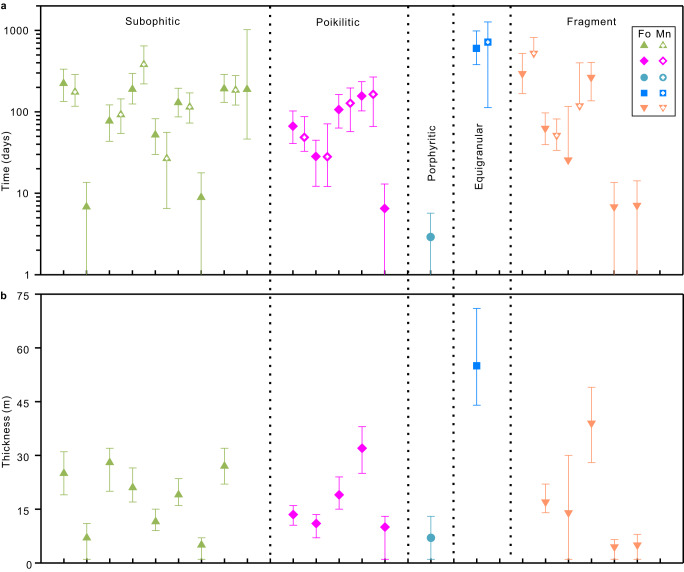
Timescales of lava cooling determined from olivine crystals and the corresponding estimated thicknesses. **a** Histogram showing the distribution of timescales modeled from olivine Fo and Mn. The times are shown in each olivine crystal. The diffusion model parameters and results are provided in Supplementary Data 3. The error bars shown here are calculated from DIPRA and correspond to the analytical uncertainty and a temperature uncertainty of ±30 °C (Supplementary Table 1). Abbreviations: Fo forsterite, Mn Manganese. **b** The corresponding thickness was calculated using the timescales estimated from olivine Fo. The error bars shown here result from the uncertainty in the timescales (Supplementary Table 1).

We obtained cooling timescales of 21 olivine crystals, which are summarized in Supplementary Table 1. Mn concentrations were also modeled for most olivines. The timescales obtained from Mn for the same traverse have a larger uncertainty, but are consistent with the timescales estimated from Fo (Supplementary Table 1). For the olivine crystals of Group 1, the timescales estimated from the growth-dominated crystals range from ${2.9}_{+2.9}^{-2.8}$ to ${8.9}_{+8.9}^{-8.8}$ days. The timescales of the diffusion-dominated crystals range from ${25.6}_{+90.9}^{-26.4}$ to ${602.3}_{+382.6}^{-222.4}$ days, with ~50% of the timescales determined being longer than 3 months (Fig. 4a; Supplementary Table 1). By contrast, an olivine grain (103-025, 009#ol2) from Group 2 is composed of 3 regions with different crystallographic orientations (Supplementary Fig. 9a). However, there are no obvious grain boundaries between these three regions in the BSE image (Supplementary Fig. 9b), suggesting that they originated from the same olivine nucleation, but were subjected to compression or similar factors that eventually changed orientation. During later growth, the more central region (region B marked in Supplementary Fig. 9b) probably formed a reverse zonation because it has a higher Mg content on both sides (Supplementary Fig. 9c). In addition, we also calculated a diffusion time of ${188.7}_{+831.1}^{-142.4}$ days (Supplementary Table 1). Finally, it should be noted that the timescale obtained corresponds to a lower estimate when considering the fixed temperature used in the modeling. Our data set of timescales provides first-order information on the timing of cooling events.

### Estimated thickness of basaltic lava flow

Unlike the Apollo samples, which contained the products of multiple volcanic eruptions (i.e., basalts of different ages were found at each Apollo landing site), the Chang’e-5 basalts are most likely from a single eruptive event (see discussion text) and therefore provide an unprecedented window into the thickness of mare basalts. We used COMSOL Multiphysics software (www.comsol.com), which includes the one-dimensional heat conduction equation, to model heat transfer from lunar lava flows to the underlying particulate regolith. In the absence of an atmosphere, the top surface of the lava cools by radiation to space, while the base cools by conduction to the regolith/basalt below. In our model, the initial temperature, density, specific heat capacity, and thermal conductivity are the most important parameters for both the overlying lava flow and the underlying material^31^. Here, the initial temperature of the lunar lava flow is set at 1200 °C based on the modeling result^32^, and the density of the erupted magma (~2940 kg/m^3^) is derived from the PETROLOG program^33^. The heat capacity and thermal conductivity for the hot lava flow are assumed based on a previous study^31^. According to the study of ref. ^34^, the Em4/P58 unit of the Chang’e-5 landing site directly overlies the Em3 basalt unit instead of regolith. As such, the cold basalt is used as the underlying component, although we cannot completely exclude the possibility of the presence of regolith. We assume that the parameters for this cold basalt are the same as those used in ref. ^35^. The cooling curve is a function of basalt thickness and time. The thickness of the Chang’e-5 basalt lava flow can be inferred from the timescales and temperatures of the olivine crystals. The uncertainty in the thickness is estimated based on the uncertainties in the timescales and temperatures of the olivine crystals. The basalt thicknesses estimated from individual olivine crystals are shown in Fig. 4b. They vary from ${4.5}_{+2}^{-3.5}$ to ${55}_{+16}^{-11}\,{{{{{\rm{m}}}}}}$, with most ranging from 8 to 30 m. Assuming that all soil clasts studied are from the same lava flow, we can derive an average value of ${18.5}_{+5.5}^{-6}\,{{{{{\rm{m}}}}}}$, which is probably the minimum value for lava flow thickness. The thickness of unit Em4/P58, where Chang’e-5 landed, was previously estimated to be 39.1–62.7 m based on remote observations^36^. The value we estimate is largely consistent with the estimated thickness from remote observations.

## Discussion

The identical Sr-Nd-Pb isotopic compositions in all analyzed basalts (ref. ^6,10,37^) indicate that the Chang’e-5 basalts originated from the same source, and the high-precision U-Pb dating of basalt clasts of different textures^5,6^ suggests that they formed in a single eruptive event. Furthermore, almost all olivine and clinopyroxene crystals from the 26 basalt clasts (Fig. 2), in combination with clinopyroxene from the literature^10^, resemble those from the Apollo and Luna collections, of which only a few lunar samples have been reported to have oscillatory zonation that could record compositional and/or state changes (e.g., lunar basalt 12021, NWA 032; refs. ^38–40^). The normal zoning patterns in the Chang’e-5 basalts exclude the incorporation of exogenous material during the formation of these basalts, which agrees well with the inference derived from the Sr-Nd-Pb isotopes of basalt clasts^5,6,10^. In a summary, the Chang’e-5 basalts record a relatively simple evolutionary history without remarkable magma replenishment or crustal assimilation during their formation.

**Fig. 5:**
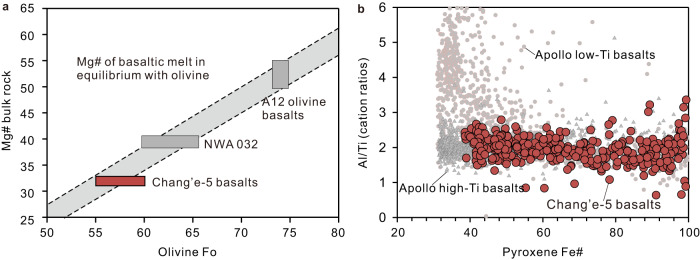
Chemical composition of the bulk rock, olivine and pyroxene from the Chang’e-5 basalts. **a** Olivine Fo vs. bulk-rock Mg# [defined as Mg/(Mg + Fe) × 100, mole fraction]. The gray area was constructed using olivine-basaltic melt (*K*_*d*_^Fe-Mg^_Ol-Melt_ = 0.33; ref. ^53^). Olivine from Apollo 12 basalts and meteorite NWA 032 are plotted for comparison^59^. The Chang’e-5 basalts are thought to have a similar source to the Apollo 12 basalts. The Mg# of the bulk rock is from ref. ^10^. Abbreviation: Fo, forsterite. **b** Al/Ti vs. pyroxene Fe# [defined as Fe/(Fe + Mg) × 100, mole fraction]. Data for Chang’e-5 pyroxene are from refs. ^5, 9, 10^. The Apollo low-Ti and high-Ti basalts are from a MoonDB search (http://search.moondb.org/).

Using the exchange coefficients, the cores of olivine crystals in the Chang’e-5 basalts are nearly in equilibrium with the bulk-rock composition (Fig. 5a), indicating that most olivine grains crystallized directly from the parental melt in a closed-system.

The absence of megacrysts derived from the deep magma chamber is consistent with the rarity of xenoliths and megacrysts found in lunar materials returned by the Apollo and Luna missions or in lunar meteorites^41^. Based on trace element abundances in augite, the degree of fractional crystallization prior to eruption was estimated to be >40% (ref. ^10^), which is generally greater than samples returned by the Apollo missions (<30%; ref. ^42^). After the eruption, extensive fractional crystallization also occurred during cooling of the lava flow. Euhedral spinel observed as inclusions in olivine and clinopyroxene (Supplementary Figs. 1–6) indicate an early crystallization phase. Augite and plagioclase may have simultaneously crystallized, as suggested from the Chang’e-5 pyroxenes all displaying similar Al/Ti ratios (~2; Fig. 5b). Differentiation of lava flow would enable the formation of a cumulative base and facilitate variable cooling within the stratigraphy, which would explain the different textures and modal mineralogies of the Chang’e-5 mare basalts. From the different average timescales for the different textures (Supplementary Table 1), the stratigraphic succession could be inferred, with porphyritic basalts probably originating from the rapidly cooled upper or lower part of the flow, subophitic and poikilitic from the more slowly cooled middle part of the flow, and the equigranular basaltic fragments from the center of the lava flow (Fig. 4). Similar textural sequences have also been proposed for lunar basaltic meteorite NWA 032 (porphyritic texture) and LaPaz (subophitic texture)^22^.

**Fig. 6:**
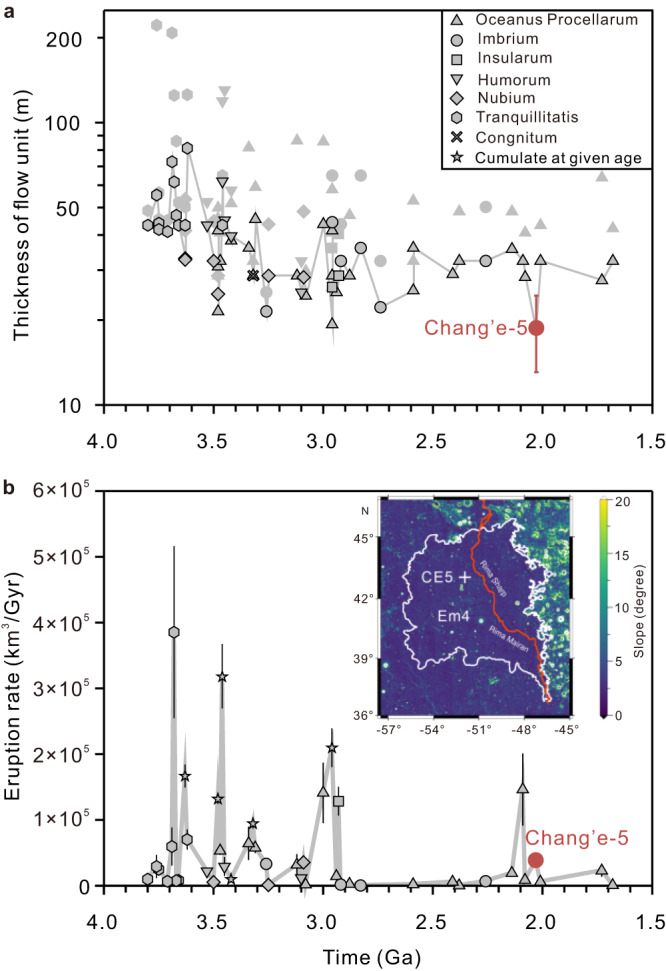
Variations in mare volcanism throughout time. **a** The estimated thicknesses of basaltic lava flows from the nearside basins, including Oceanus Procellarum, Imbrium, Tranquillitatis, Humorum, Congnitum, Nubium, and Insularum (ref. ^12^), are shown as minimum (black outline) and maximum (gray outline) values. The error bars for the Chang’e-5 samples represent the uncertainty in the average thickness. The vertical axis is a logarithmic scale. **b** Eruption rates are calculated based on the volume of mare basalts at a given time and the interval between 2 close eruptions. Original data are from ref. ^12^. and this study. The error bars are calculated from the uncertainties in lava flow thickness. The inset map shows the terrain slope of the Em4 unit and the position of the Chang’e-5 landing site. The average slope of the Em4 unit is about 3.3°, indicating a relatively flat terrain. Abbreviations: CE5 Chang’e-5, Em4 Eratosthenian-aged mare unit 4, Ga Giga annum.

Our estimation of the minimum thickness of the Chang’e-5 basalt flow is near the lower end of the range estimated for individual mare basalt flow units on the nearside basins that erupted between 3.8 and 1.5 Ga (Fig. 6a). Furthermore, the terrain of Em4/P58 unit is relatively flat, with an average slope of 3.3° (inset map in Fig. 6b). If we assume that the Em4/P58 unit is covered by uniformly thick basalts, this results in a large volume for the Chang’e-5 basalt flow (~473–894 km^3^), and thus provides direct evidence for a huge volume of the late-stage volcanic eruptions. This result suggests that although the frequency of magmatic eruptions decreases with lunar age, individual young eruption events can have a high volume flux, at least locally. We find that there was an enhancement of volcanic eruption flux about 2.0 billion years ago (Fig. 6b), concentrated mainly in the Oceanus Procellarum basin. This suggests that volcanic activity on the Moon did not decrease monotonically with time, but that episodic eruptions continued to occur during the late stage of the Moon. This uptick in the flux of magma eruption has been proposed in previous studies based on remote sensing data. Du et al.^43^ calculated the eruption rate of mare basalts based on the morphology of partially buried craters, where a peak in the eruption rate was found to occur at 1.7 Ga. Hiesinger et al.^13^ and Morota et al.^14^ dated basaltic units on the nearside maria using the crater-counting chronology, and showing enhanced volcanic activity occurring during 1.8–2.2 Ga ago. The increased volcanic activity was not caused by the presence of KREEP in the mantle source^10^, but rather may have been driven by fusible components in the mantle source^33^, tidal heating from Earth^5^, and/or lunar mantle convection cycles^44^. There are at least two favorable conditions to trigger the volcanism. One is that the crust beneath the Oceanus Procellarum basin is very thin compared to other regions (typically <30 km versus 34–43 km; ref. ^45^). The second condition could be that there are large-scale, deep-seated fractures in the crust caused by the formation of the multi-ring system of the Imbrium basin^46^, which are favorable for magma ascent through the lunar crust^47^.

## Methods

### Sample description

The Chang’e-5 basalt clasts studied in this work are from three soil samples (CE5C0100YJFM00103 ~1000 mg, CE5C0400YJFM00406 ~2000 mg, and CE5Z0303YJ ~200 mg). Rock fragments (0.4–3.5 mm in size) from these three soils were collected in an ultra-clean laboratory housed at the Institute of Geology and Geophysics, Chinese Academy of Sciences (IGGCAS). The fragments of interest were embedded in epoxy resin and polished for in situ chemical and isotopic analyses. Before analysis, the epoxy mounts were stored in a vacuum drying oven. We also analyzed olivine and pyroxene grains from 9 basalt clasts whose petrographic texture and geochronology were previously reported^6,10^.

### Scanning electron microscope

The textural and morphological features of the basalt clasts were characterized using a Thermo Scientific Apreo scanning electron microscope (SEM) at the IGGCAS. The epoxy mounts of the samples were coated with ~20 nm carbon. An electron beam with 15 kV accelerating voltage and 6.4 nA beam current was chosen to obtain high-resolution images.

### Electron microprobe analysis

Major elements (Mg, Fe, and Si) and minor elements (Mn, Al, and Ca) in olivine grains from the Chang’e-5 basalt clasts were measured using a Cameca SXFive EMPA at the IGGCAS. The analytical conditions were an accelerating voltage of 25 kV and a beam current of 400 nA, and the beam size was about 5 μm. Analytical crystals and on-peak count times were used for the high-precision work as follows: Si (TAP, 10 s), Mg (TAP, 10 s), Fe (LLIF, 10 s), Ca (LPET, 60 s), Mn (LIF, 60 s), and Al (TAP, 180 s). The MongOl Sh11-2 olivine was used as a standard and analyzed five times per 50–60 measurements to monitor instrument drifts. The analytical errors for Mg, Fe, Si, Mn, Al, and Ca were tested by repeated analyses of MongOl Sh11-2 olivine (see Supplementary Data 4 for more details). The detection limits are 39–63 ppm for Mn, 14–23 ppm for Al, and 12–19 ppm for Ca. The corresponding standard errors are ~50 ppm, ~16 ppm, and ~17 ppm for Mn, Al, and Ca, respectively. X-ray element maps of Fe, Mn, and Ca were collected in selected olivine crystals using a voltage of 20 kV, a beam of 200 nA, a resolution of 1.0 µm, and a dwell time of ~100 ms/pixel. The working conditions for the Al X-ray map were as follows: a voltage of 20 kV, a beam of ~350 nA, a resolution of ~0.5 µm, and a dwell time of ~200 ms/pixel. In addition, a JEOL JXA8100 electron probe was used to measure the chemical composition of clinopyroxene grains in selected Chang’e-5 basalt clasts. Point analyses were performed under the following conditions: an accelerating voltage of 15 kV, a beam current of 20 nA, ~1 μm beam diameter, and a total time of ~3.5 min for each point. On-peak count times were 10 s for Mg, Fe, Cr, and Ca, and background count times were 5 s for Mg, Fe, Cr, and Ca. Natural and synthetic standards were measured during the analyses, and matrix corrections were based on ZAF procedures.

### In situ mineral orientations

Olivine crystallographic axes were determined using a Nova NanoSEM 450 field emission SEM equipped with an Oxford/HKL EBSD system, also at the IGGCAS. Prior to measurements, we use a BUEHLER VibroMet 2 vibratory polishing machine to remove surface stress. The working conditions for the EBSD analyses were 20 kV, a spot size of 6, and a tilt angle of 70°. The area with obvious diffraction patterns was selected for automatic beam scanning. The data were then plotted on a stereographic projection of the lower hemisphere, and the angle between the orientations of the three axes and the traverses of the electron microprobe was measured using HKL software. The EBSD results are included in Supplementary Data 1.

### Diffusion modeling

Concentration gradients (e.g., Fe-Mg, Ca, Ni) in olivine were modeled using the DIPRA software program^48^, with applications to determine the timescales of magmatic processes^29^. This software was developed based on a finite difference scheme, enabling simultaneous modeling of the diffusion of many elements, as mentioned above. It also accounts for uncertainties arising from chemical composition and temperature^48^. The one-dimensional diffusion equation used in the DIPRA program is written as $\frac{\partial {\theta }_{i}}{\partial t}=\frac{d{D}_{i}}{\partial {\theta }_{{Fo}}}\frac{\partial {\theta }_{{Fo}}}{\partial x}\frac{\partial {\theta }_{i}}{\partial x}\,+{D}_{i}\frac{{\partial }^{2}{\theta }_{i}}{{\partial }^{2}x}$ (where *i* is Fo, Mn, Ni, or Ca; *θ*_*Fo*_ is the concentration of Fo; *D* is the diffusion coefficient) (see ref. ^48^. for more details). The cation diffusivities used in the program are experimentally determined data that depend on oxygen fugacity, crystallographic direction, composition, and temperature^49–51^. The diffusivity of Fe-Mg in olivine along the [001] direction is log[D_FeMg_(*m*^2^*/s*)] = −9.21 − [(*E*_*Fo*_ + (*P* − 10^5^) × 7 × 10^−6^]/2.303RT + 1/6 × log(*fO*_*2*_/10^−7^) + 3(0.9 − *X*_*Fo*_), where *P* refers to pressure in units of Pa, *R* = 8.314 J mol^−1^K^−1^, *T* refers to absolute temperature, *E*_*Fo*_ is the activation energy, and X_Fo_ refers to the mole fraction of forsterite^51^. The expressions for Mn and Ni are log[D_Mn_(*m*^*2*^*/s*)] = −9.21 − [(*E*_*Mn*_ + (*P*−10^5^) × 7 × 10^−6^]/2.303RT + 1/6 × log(*fO*_*2*_/10^−7^) + 3(0.9 − *X*_*Fo*_) and log[D_Ni_(*m*^*2*^*/s*)] = −8.41 − [(*E*_*Ni*_ + (*P* − 10^5^) × 7 × 10^−6^]/2.303RT + 1/4.25 × log(*fO*_*2*_/10^−6^) + 1.5(0.9 − *X*_*Fo*_), respectively^48^. Iron, Mg, Mn, and Ni in olivine show similar features of diffusion anisotropy, and diffusion along [001] is about 6 times faster than along [100] or [010], i.e., $D\frac{{Fe}-{Mg}}{[001]} \sim 6D\frac{{Fe}-{Mg}}{[010]} \sim 6D\frac{{Fe}-{Mg}}{[100]}\,$(refs. ^51,52^). Thus, the $D$ along the direction of the electron microprobe traverse depends on its orientation with respect to the crystallographic axes of olivine. Moreover, Mg-Fe diffusion at magmatic temperatures can change from the TaMED (transition metal extrinsic domain) mechanism (dependent on *fO*_*2*_) to the PED (purely extrinsic domain) mechanism (independent of *fO*_*2*_) at lower temperatures^52^. In this work, we measured the crystallographic axes of olivine using EBSD and the angles between the electron microprobe traverse and the three axes (Supplementary Data 1). Using the PETROLOG software modeling results^33^ and the Fe-Mg exchange partition coefficient between olivine and basaltic melt (*K*_*d*_^Fe-Mg^_Ol-Melt_ = 0.33; ref. ^53^), we estimated the average liquidus temperature for each olivine grain based on its Fo content (Supplementary Table 1). Given the many unknown factors in the melt, a conservative uncertainty of ±30 °C was assumed in this model. Based on previous studies^54,55^, the oxygen fugacity (log *f*O_2_) for lunar basalt is equal to the NNO buffer minus 5.5 log units.

For the diffusion-dominated olivine grains, we note that the Al concentration within the uncertainty is almost identical from the core to the rims. In this case, we assume that the “rim” is very thin. Then, we used the measured Fo value at the center of the grain as an initial value for the whole grain, except at the grain boundary, where we used the measured Fo value at the rim as the initial value for the boundary (Supplementary Data 3). Fifteen olivine crystals showed simple Fo zoning and decoupled Al-Fo signatures that were modeled with a single timescale. Manganese concentrations were also modeled to see if the timescales for each crystal were concordant. Despite the possibility that growth plays a minor role, the consistent timescales obtained from Fo and Mn (Fig. 4a) suggest that the observed Al-Fo decoupling is driven by diffusion rather than growth^56^.

For individual olivine grains dominated by growth, we use the size of the olivine grain and the growth rate to calculate the timescales. Since the exact growth rate of olivine under lunar conditions was not known, we used the experimentally determined growth rate from 10^−6^ to 10^−10^ m/s to estimate the timescale (refs. ^57,58^). The entire range was used to estimate the minimum and maximum growth time for olivine. All cooling timescale results are summarized in Supplementary Table 1.

## Supplementary information

Supplementary Information

Description of Additional Supplementary Files

Supplementary Data 1

Supplementary Data 2

Supplementary Data 3

Supplementary Data 4

## Data Availability

All data generated or analyzed during this study are included in this published article and supplementary information files. For the data policy, all of the data above for this paper are also available in Figshare (https://figshare.com/articles/dataset/21875373). Source data are provided with this paper.

## Source data

Source Data

## Code availability

The diffusion model code (software) used in this work is available at http://www.tgirona.com/.
