# Supplementary material for: Surges in volcanic activity on the Moon about two billion years ago
Source: Nat Commun. 2023 Jun 22;14:3734. doi: 10.1038/s41467-023-39418-0 (PMC10287643; doi:10.1038/s41467-023-39418-0)
Supplement: Supplementary file 1 — Supplementary Information [file 41467_2023_39418_MOESM1_ESM.pdf]

Supplementary Materials

Surges in volcanic activity on the Moon about two billion years ago

Heng-Ci Tian[1]*, Chi Zhang[1]*, Wei Yang[1], Jun Du[2], Yi Chen[3], Zhiyong Xiao[4], Ross N. Mitchell[3], Hejiu Hui[5], Hitesh G. Changela[1,6], Tian-Xin Zhang[7,8], Xu Tang[1], Di Zhang[3], Yangting Lin[1], Xianhua Li[3], Fuyuan Wu[3]

[1] Key Laboratory of Earth and Planetary Physics, Institute of Geology and Geophysics, Chinese Academy of Sciences, Beijing 100029, China
[2] National Space Science Center, Chinese Academy of Sciences, Beijing 100190, China
[3] State Key Laboratory of Lithospheric Evolution, Institute of Geology and Geophysics, Chinese Academy of Sciences, Beijing 100029, China
[4] Planetary Environmental and Astrobiological Research Laboratory, School of Atmospheric Sciences, Sun Yat-sen University, Zhuhai 519080, China
[5] State Key Laboratory of Mineral Deposits Research and Lunar and Planetary Science Institute, School of Earth Sciences and Engineering, Nanjing University, Nanjing 210023, China
[6] School of Mining and Metallurgical Engineering, National Technical University of Athens, Athens, Greece
[7] Deep Space Exploration Laboratory, Beijing 100043, China
[8] Lunar Exploration and Space Engineering Center, China National Space Administration, Beijing 100190, China

Contacting email: hctian@mail.iggcas.ac.cn (H.C.T); czhang@mail.iggcas.ac.cn (C.Z.)

This PDF file includes:

Supplementary Figures 1-9
Supplementary Table 1

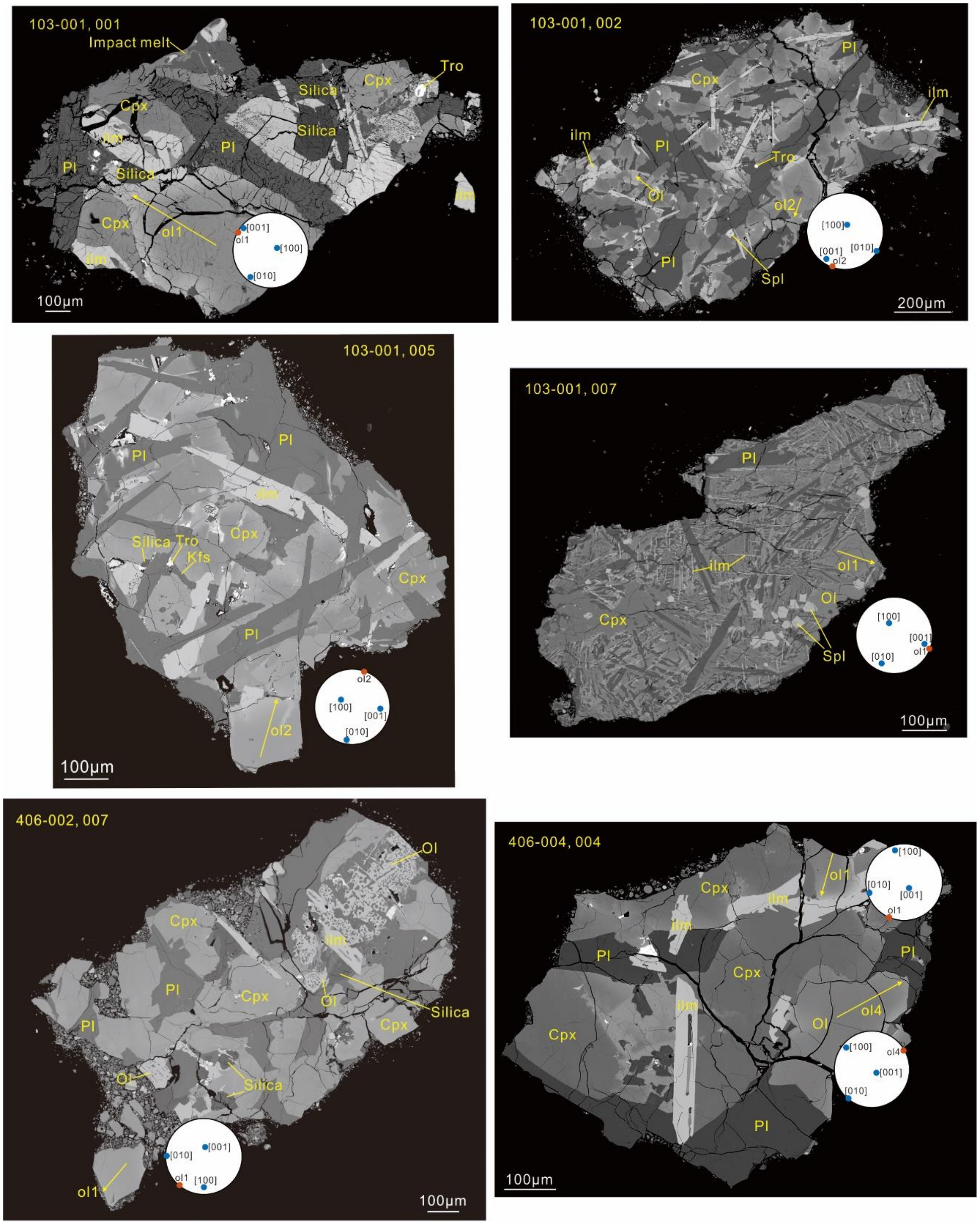

Supplementary Fig. 1. BSE images of the Chang’e-5 basalts (103-001, 001; 103-001, 002; 103-001, 005; 103-001, 007; 406-002, 007; 406-004, 004). The yellow lines with arrows mark the positions of the EMPA traverses (data provided in Supplementary Data 1). EBSD results showing the crystallographic and traverse orientation projected on the lower hemisphere. Abbreviation: Ol, olivine; Pl, plagioclase; Cpx, clinopyroxene; ilm, ilmenite; Tro, troilite; Spl, spinel; Kfs, K-feldspar.

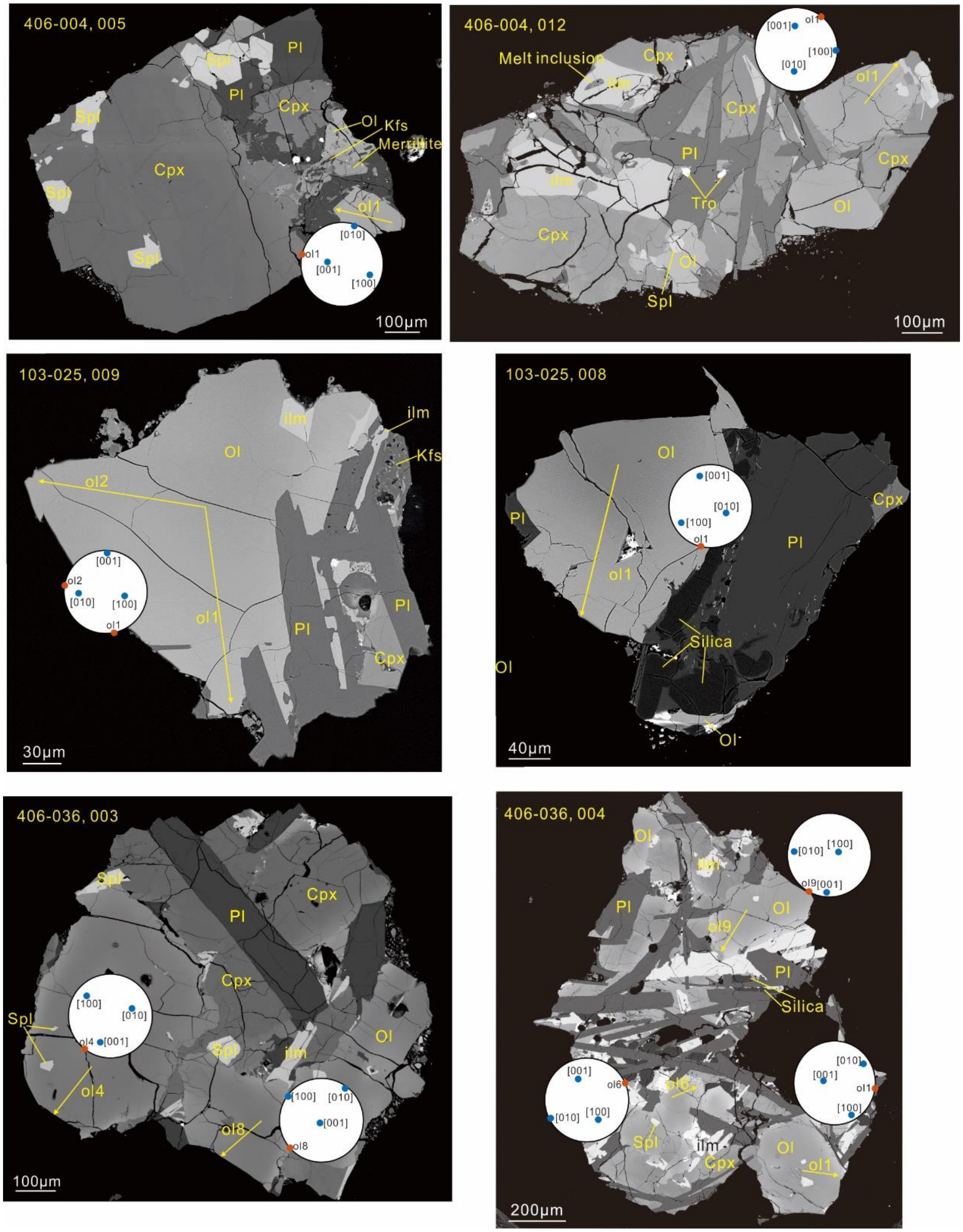

Supplementary Fig. 2. BSE images of the Chang'e-5 basalts (406-004, 005; 406-004, 012; 103-025, 009; 103-025, 008; 406-036, 003; 406-036, 004). The yellow lines with arrows mark the positions of the EMPA traverses (data provided in Supplementary Data 1). EBSD results showing the crystallographic and traverse orientation projected on the lower hemisphere. Abbreviation: Ol, olivine; Pl, plagioclase; Cpx, clinopyroxene; ilm, ilmenite; Tro, troilite; Spl, spinel; Kfs, K-feldspar.

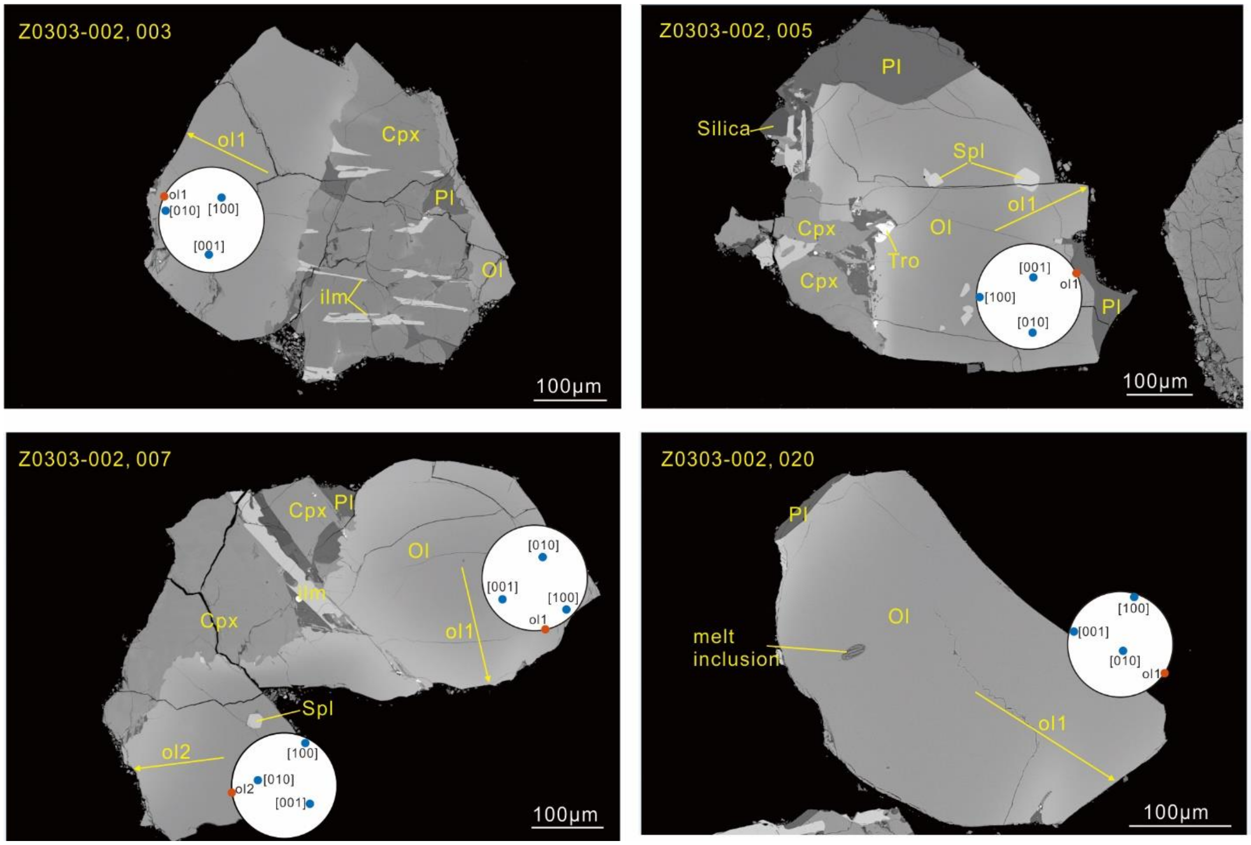

Supplementary Fig. 3. BSE images of the Chang'e-5 basalts (Z0303-002, 003; Z0303-002, 005; Z0303-002, 007; Z0303-002, 020). The yellow lines with arrows mark the positions of the EMPA traverses (data provided in Supplementary Data 1). EBSD results showing the crystallographic and traverse orientation projected on the lower hemisphere. Abbreviation: Ol, olivine; Pl, plagioclase; Cpx, clinopyroxene; ilm, ilmenite; Tro, troilite; Spl, spinel.

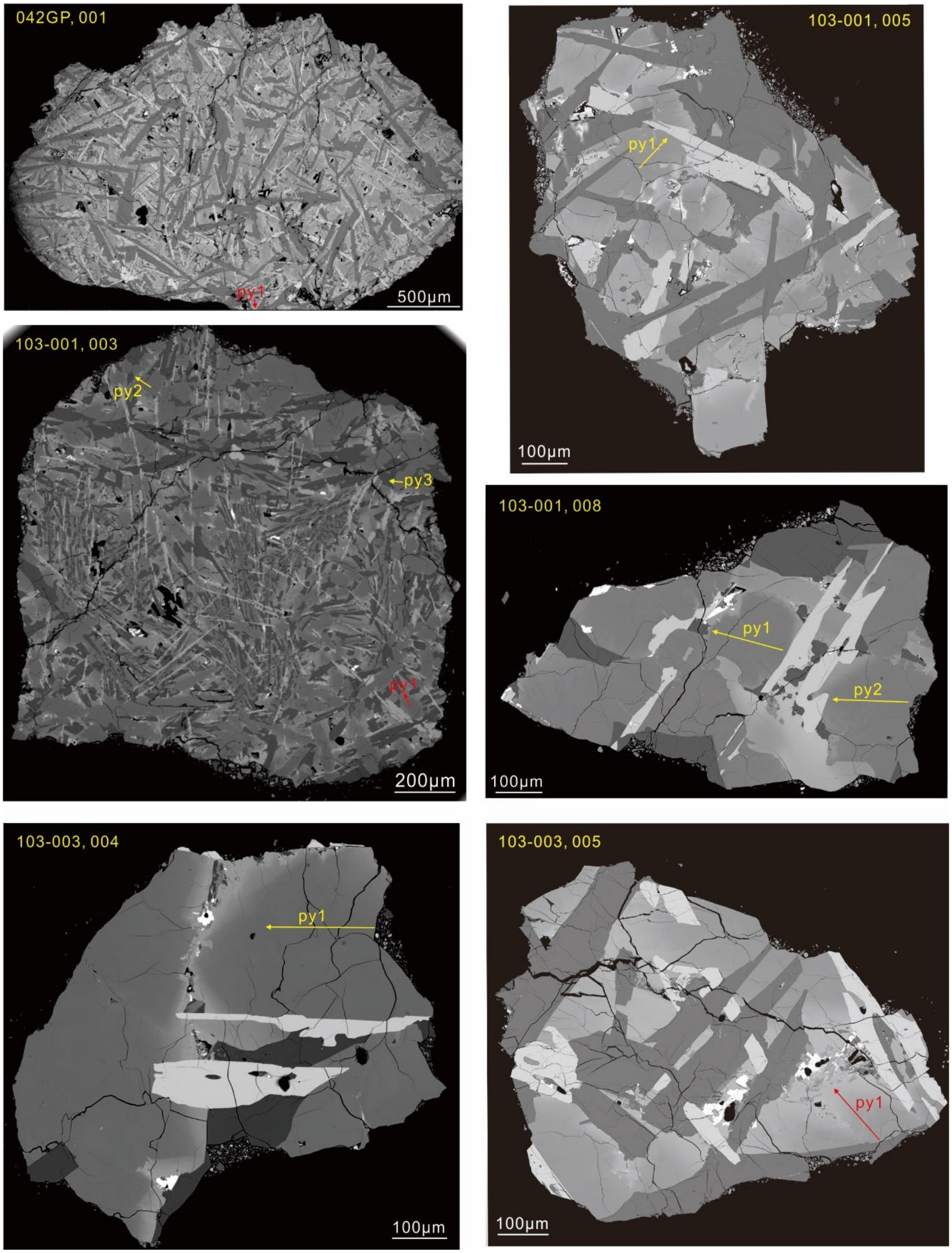

Supplementary Fig. 4. BSE images of the Chang'e-5 basalts (042GP, 001; 103-001, 005; 103-001, 003; 103-001, 008; 103-003, 004; 103-003, 005). The red lines with arrows mark the positions of the clinopyroxene EMPA traverses from Tian et al. (2021), and the yellow lines with arrows mark the positions of the clinopyroxene EMPA traverses from this work (Supplementary Data 2).

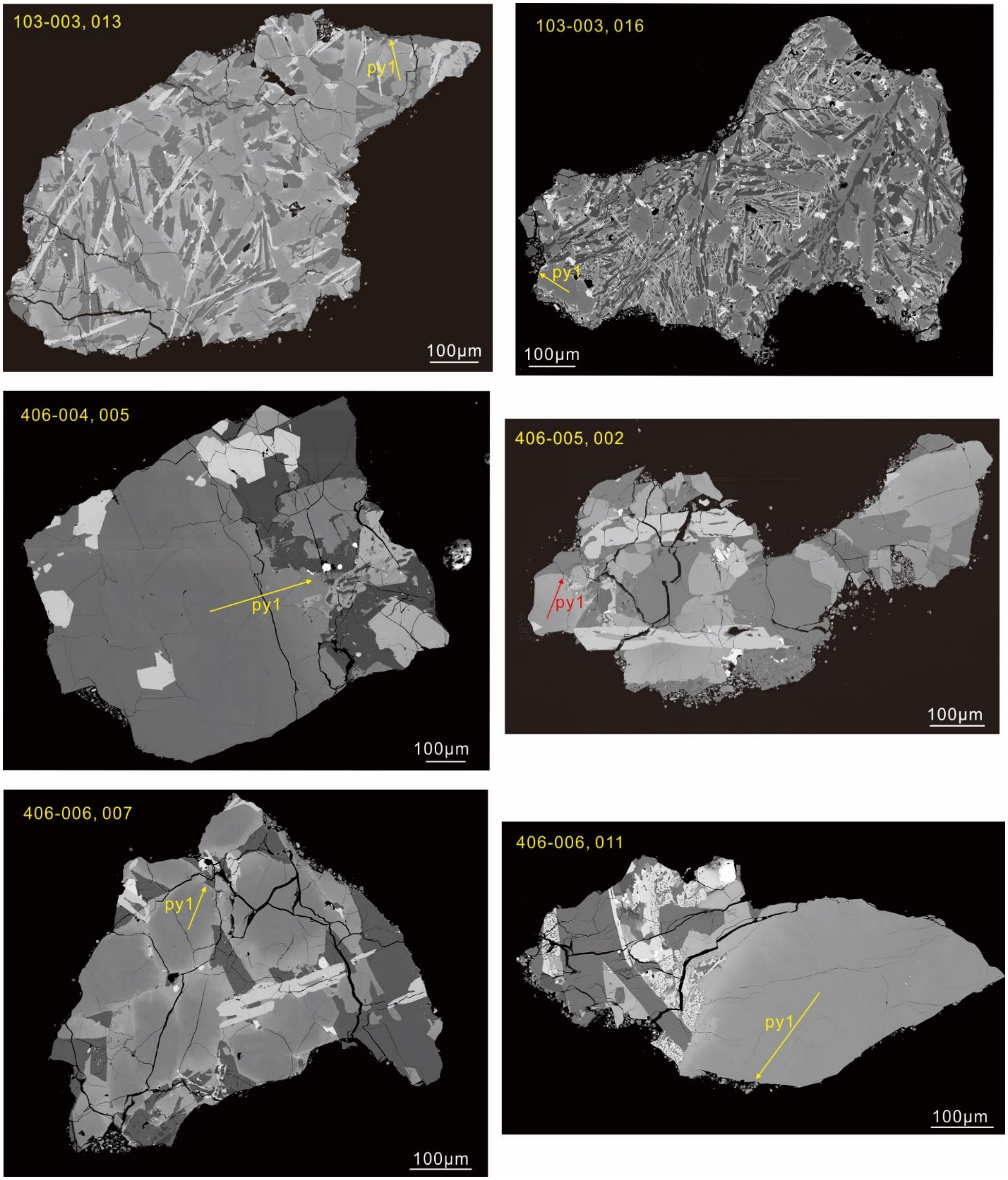

Supplementary Fig. 5. BSE images of the Chang'e-5 basalts (103-003, 013; 103-003, 016; 406-004, 005; 406-005, 002; 406-006, 007; 406-006, 011). The red lines with arrows mark the positions of the clinopyroxene EMPA traverses from Tian et al. (2021), and the yellow lines with arrows mark the positions of the clinopyroxene EMPA traverses from this work (Supplementary Data 2).

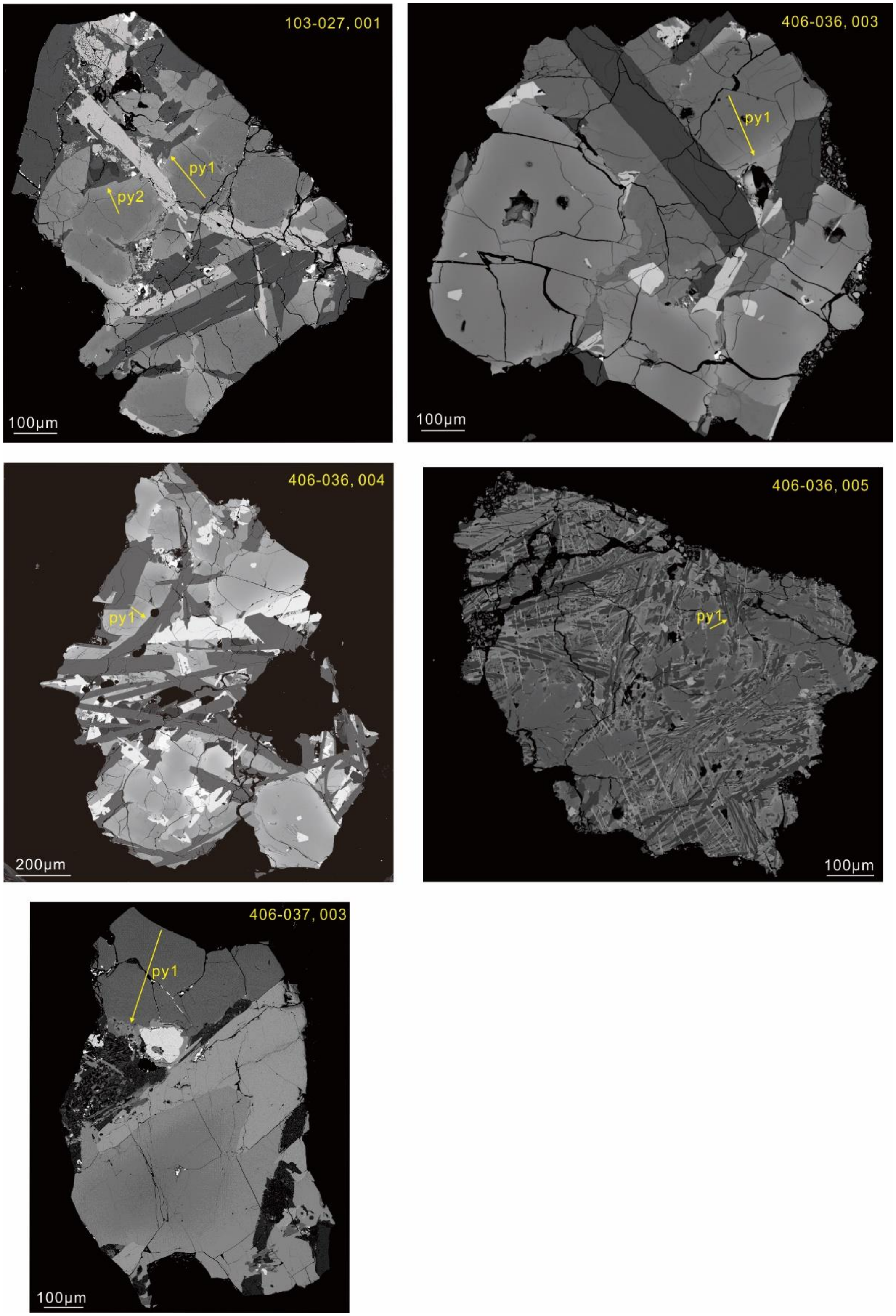

Supplementary Fig. 6. BSE images of the Chang’e-5 basalts (103-027, 001; 406-036, 003; 406-036, 004; 406-036, 005; 406-037, 003). The yellow lines with arrows mark the positions of the clinopyroxene EMPA traverses from this work (Supplementary Data 2).

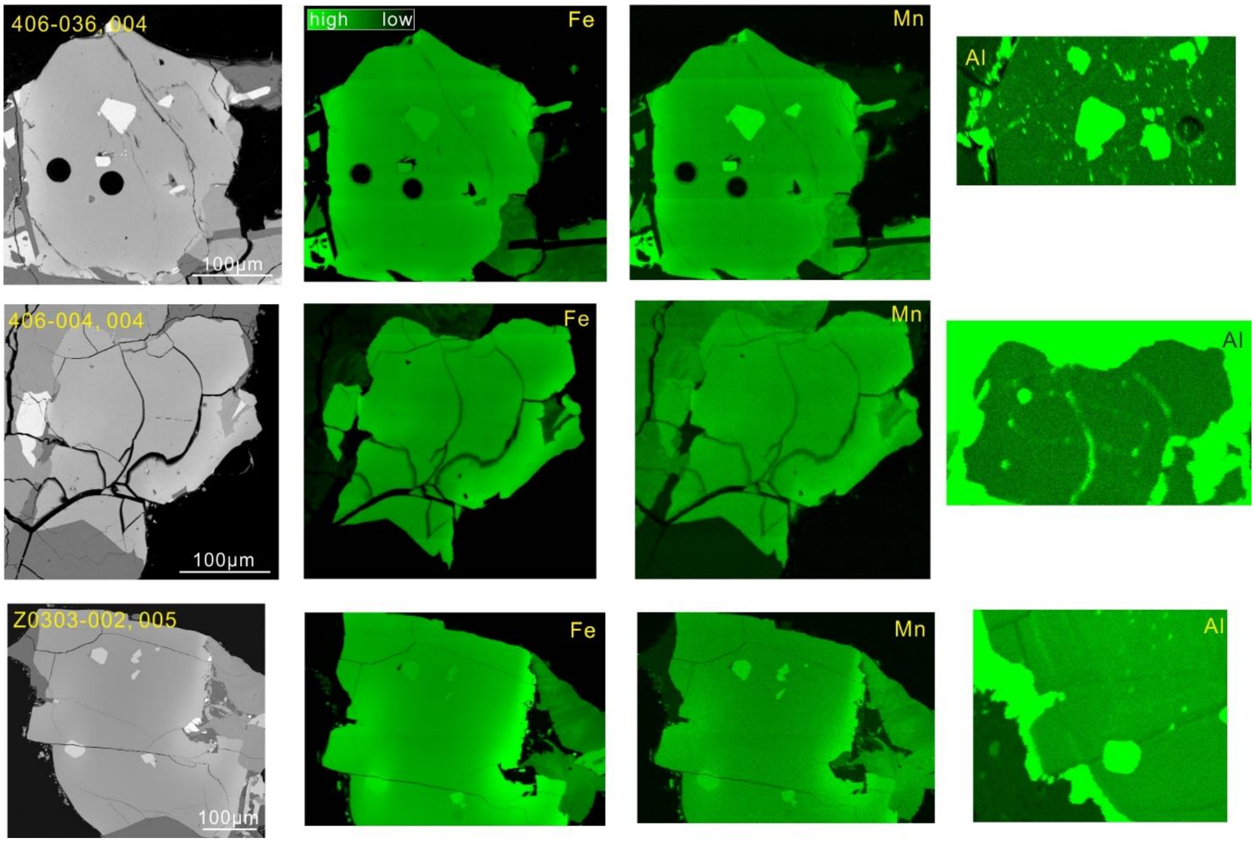

Supplementary Fig. 7. BSE images and the Fe, Mn, and Al X-ray maps of the selected olivine crystals from the Chang'e-5 samples (406-036, 004; 406-004, 004; Z0303-002, 005). Abbreviations: Fe, iron; Mn, Manganese; Al, Aluminium.

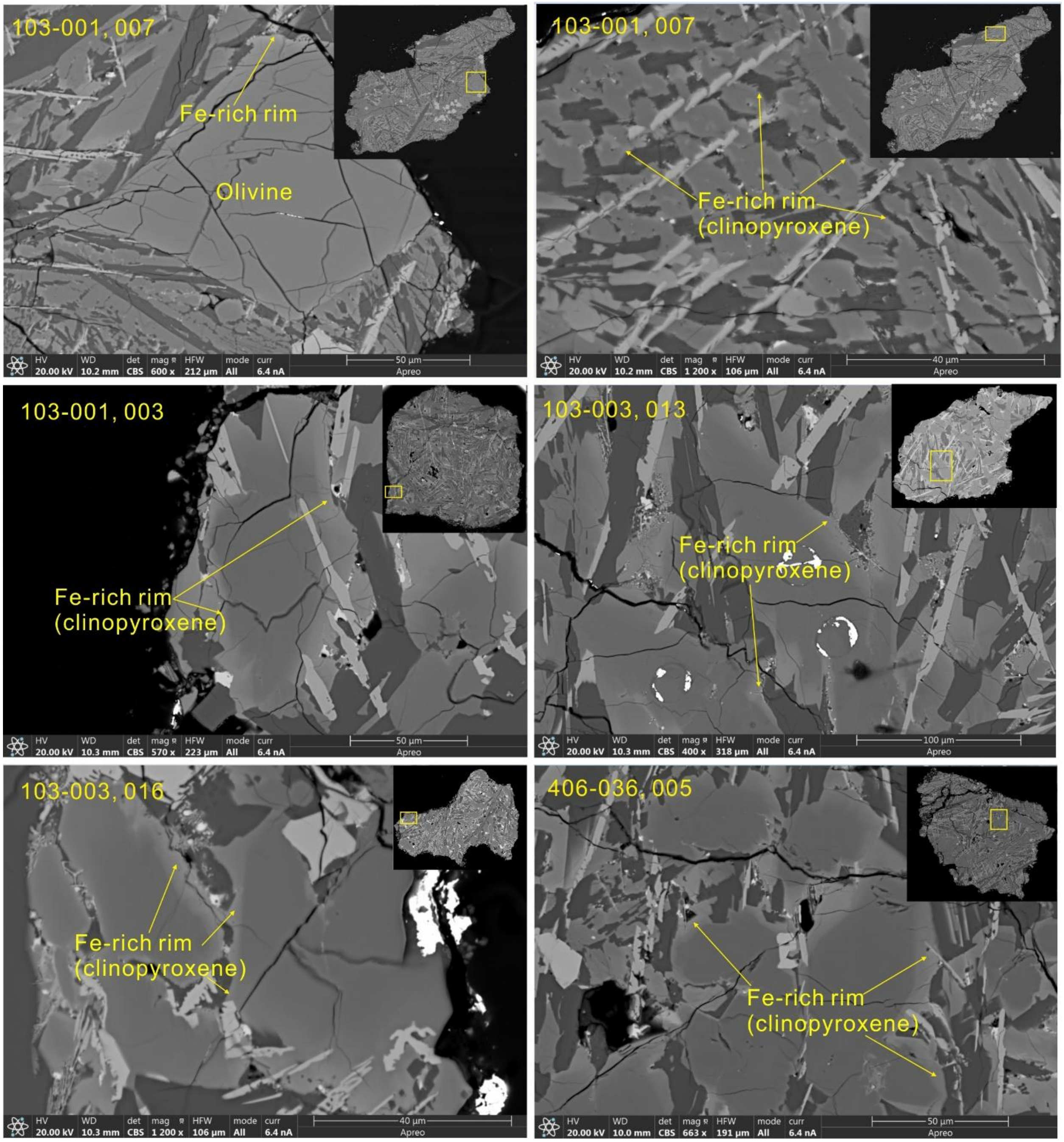

Supplementary Fig. 8. The BSE images show the distribution of Fe-rich rims around olivine and clinopyroxene crystals from Chang’e-5 basalt clasts that exhibit porphyritic texture. The yellow rectangles mark the positions.

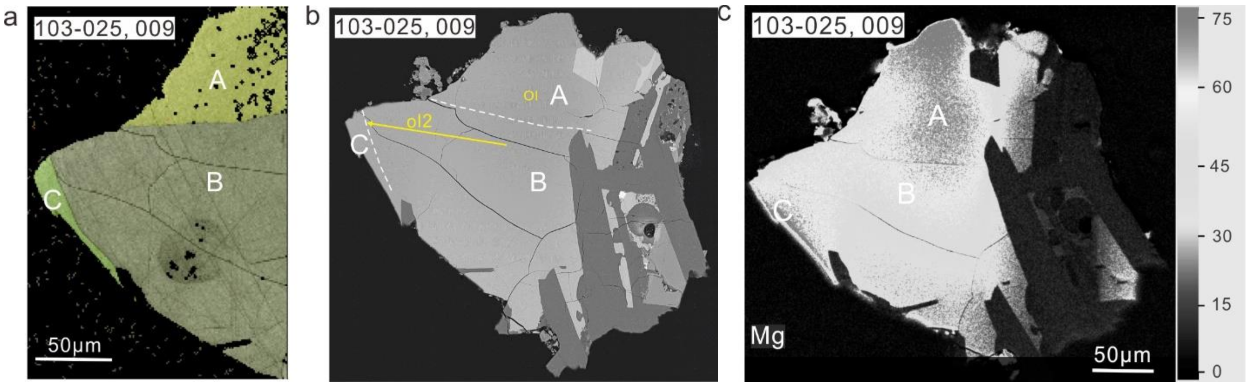

Supplementary Fig. 9. Electron backscatter diffraction (EBSD) image (a), BSE image (b), and energy dispersive spectrometry (EDS) map (c) for olivine grain from the Chang'e-5 basalt 103-025, 009. The olivine grain is divided into three regions based on the crystal orientation shown in the EBSD map. Regions A and C have the same crystal orientation but are different from region B. However, there are no cracks or boundaries between A and B or between B and C, indicating that the three regions likely originated from the same olivine nucleation rather than from three independent grains. Since region B has a lower Mg content than regions A and C, this suggests that region B crystallised relatively late and was disturbed or compressed by an external factor. Consequently, this likely resulted in a change in orientation and the formation of a reverse zoning (traverse ol2) between A and C during later growth. Abbreviation: Ol, olivine.

Supplementary Table 1. Summary of olivine and clinopyroxene crystals, estimated timescales and lava thicknesses in this work.

Sample	Crystal type	Texture	Zoning	Traverse distance (µm)	Mg# range (EMPA)	Type	Average Temperature (°C)	Time from Fo (days, error)	Time from Mn (days, error)	Thickness (m)
Olivine										
103-001, 005#ol2	Group 1	Subophitic	normal	120.9	54.4-29.5	Diffusion-dominated	1095	223.1 (-89.1, +111)	174.9 (-57.7, +112.3)	25 (-6, +6)
406-004,012#ol1	Group 1	Subophitic	normal	117	48-37.9	Growth-dominated	1091	6.8 (-6.7, +6.8)		7 (-6, +4)
103-025,009#ol1	Group 1	Subophitic	normal	133.3	36.8-17.1	Diffusion-dominated	1070	77.2 (-33.7, +44.5)	92.3 (-38.1, +51.9)	28 (-8, +4)
406-036,004#ol1	Group 1	Subophitic	normal	130.1	59.5-39.5	Diffusion-dominated	1109	189.2 (-64.2, +107.8)	381.4 (-161.3, +263.1)	21 (-4, +5.5)
406-036,004#ol6	Group 1	Subophitic	normal	61.3	57.4-36.6	Diffusion-dominated	1103	52.1 (-22.2, +30.1)	26.7 (-20.2, +29.2)	11.5 (-2.5, +3.5)
406-036,004#ol9	Group 1	Subophitic	normal	151.4	56.8-35.2	Diffusion-dominated	1099	129.8 (-43.2, +64.9)	114.6 (-41.8, +56.2)	19 (3, +4.5)
Z0303-002, 007#ol1	Group 1	Subophitic	normal	153	51.5-21.6	Growth-dominated	1088	8.9 (-8.8, +8.9)		5 (-4, +2)
Z0303-002, 007#ol2	Group 1	Subophitic	normal	150	56.4-27.0	Diffusion-dominated	1088	192.1 (-61.5, +95.8)	184.5 (-63.4, +94.1)	27 (-5, +5)
103-001, 001#ol1	Group 1	Poikilitic	normal	330.6	55.5-20.9	Diffusion-dominated	1095	66.7 (-25.9, +35.7)	48.6 (-15.8, +38.7)	13.5 (-3, +2.5)
103-001, 002#ol2	Group 1	Poikilitic	normal	87	58.2-50.1	Diffusion-dominated	1126	28.3 (-16.1, +16.5)	28.1 (-16, +43)	11 (-4, +2.5)
406-004, 004#ol1	Group 1	Poikilitic	normal	63	54.5-38.3	Diffusion-dominated	1099	106.6 (-43.4, +56.6)	127.8 (-70.7, +68.4)	19 (-4, +5)
406-004, 004#ol4	Group 1	Poikilitic	normal	106.4	54.4-47.1	Diffusion-dominated	1112	156.6 (-53.8, +78.3)	164.1 (-98, +103.7)	32 (-7, +6)
Z0303-002, 003#ol1	Group 1	Poikilitic	normal	113	56.2-53.8	Growth-dominated	1129	6.5 (-6.4, +6.5)		10 (-9, +3)
103-001, 007#ol1	Group 1	Porphyritic	no	50.0	58.4-59.1	Growth-dominated	1146	2.9 (-2.8, +2.9)		7 (-6, +4)
406-002, 007#ol1	Group 1	Equigranular	normal	108.2	52.4-43.4	Diffusion-dominated	1102	602.3 (-222.4, +382.6)	718.1 (-605.5, +552.6)	55 (-11, +16)
406-004, 005#ol1	Group 1	Fragment	normal	140.6	30.0-23.4	Diffusion-dominated	1073	294.6 (-127.3, +226.7)	523.9 (-560.1, 296.7)	
103-025, 008#ol1	Group 1	Fragment	normal	107.3	44.3-16.0	Diffusion-dominated	1080	62.8 (-23.2, +34.2)	51.3 (-17.7, +30.4)	17 (-3, +5)
406-036, 003#ol4	Group 1	Fragment	normal	120.5	61.0-57.6	Diffusion-dominated	1145	25.6 (-26.4, +90.9)	118.5 (-144, +281.5)	14 (-13, +16)
406-036, 003#ol8	Group 1	Fragment	normal	125.8	59.7-52.0	Diffusion-dominated	1130	265 (-128.6, +139.9)		39 (-11, +10)
Z0303-002, 005#ol1	Group 1	Fragment	normal	118.2	57.4-42.0	Growth-dominated	1111	6.8 (-6.7, +6.8)		4.5 (-3.5, +2)
Z0303-002, 020#ol1	Group 1	Fragment	normal	122.7	52.9-43.7	Growth-dominated	1107	7.1 (-7.0, +7.1)		5 (-4, +3)
103-025, 009#ol2	Group 2	Subophitic	reverse	113	36.8-31.1	Diffusion-dominated	1082	188.7 (-142.4, +831.1)		
Clinopyroxene										
042GP-001#py1		Subophitic	normal	60	46.5-20					
103-001, 005#py1		Subophitic	normal	93	50.8-48.1					
406-006, 011#py1		Subophitic	no	163	53-49.7					
406-036, 004#py1		Subophitic	normal	61.7	43.4-19.4					

103-001, 008#py1	Poikilitic	normal	154	57.5-55.9
103-001, 008#py2	Poikilitic	normal	162.9	57-49.7
103-003, 005#py1	Poikilitic	normal	139.1	52.5-12.4
406-005, 002#py1	Poikilitic	normal	103	54-23.3
406-006, 007#py1	Poikilitic	normal	79	47.4-34.4
103-001, 003#py1	Porphyritic	normal	51.3	58.3-21.8
103-001, 003#py2	Porphyritic	no	90	60-59.2
103-001, 003#py3	Porphyritic	normal	52.3	59.9-56.9
103-003, 013#py1	Porphyritic	normal	96.8	53.2-36.6
103-003, 016#py1	Porphyritic	normal	86	60.4-56.7
406-036, 005#py1	Porphyritic	normal	50.4	59.1-25.9
103-027, 001#py1	Equigranular	normal	123	55.4-26.6
103-027, 001#py2	Equigranular	normal	61.2	52.9-33
103-003, 004#py1	Fragment	normal	180	54.4-51.2
406-004, 005#py1	Fragment	normal	268.4	61-39.8
406-036, 003#py1	Fragment	normal	160.4	58-21.3
406-037, 003#py1	Fragment	normal	240.8	39.1-23.7

*The timescales of the growth-controlled olivine crystals are calculated by dividing the grain size by the crystal growth rate, assuming that the growth rate remains unchanged during magma cooling. We then use the average value to represent time. The different crystals are shown in Supplementary Figs. 1-6.

* The clinopyroxene EMPA profiles are used only to determine if there is reverse zoning and a possible magma recharging process.

References cited in Supplementary materials

Tian, H. C. *et al.* Non-KREEP origin for Chang'e-5 basalts in the Procellarum KREEP Terrane. *Nature* **600**, 59-63 (2021).
