# Supplementary material for: Surges in volcanic activity on the Moon about two billion years ago
Source: Nat Commun. 2023 Jun 22;14:3734. doi: 10.1038/s41467-023-39418-0 (PMC10287643; doi:10.1038/s41467-023-39418-0)
Supplement: Supplementary file 2 — Description of Additional Supplementary Files [file 41467_2023_39418_MOESM2_ESM.pdf]

Description of Additional Supplementary Files

File Name: Supplementary Data 1
Description: The electron microprobe dataset for olivine profiles from Chang'e-5 basalt clasts.

File Name: Supplementary Data 2
Description: The electron microprobe dataset for clinopyroxene profiles from Chang'e-5 basalt clasts.

File Name: Supplementary Data 3
Description: Diffusion timescales calculated with DIPRA software from olivine profiles.

File Name: Supplementary Data 4
Description: Analytical precision of major and minor elements in olivine measured with the Cameca SXFive electron microprobe.
